# Commentary: Long COVID in pediatric age: an observational, prospective, longitudinal, multicenter study in Italy

**DOI:** 10.3389/fimmu.2025.1624011

**Published:** 2025-09-12

**Authors:** Cristiana Indolfi, Angela Klain, Giulio Dinardo, Carolina Grella, Maria Maddalena Marrapodi, Michele Miraglia del Giudice

**Affiliations:** Department of Woman, Child and General and Specialized Surgery, University of Campania ‘Luigi Vanvitelli’, Naples, Italy

**Keywords:** long COVID, children, follow-up protocol, lung ultrasound, neuropsychological symptoms

The original research entitled ‘Long COVID in pediatric age: an observational, prospective, longitudinal, multicenter study in Italy’, conducted by Esposito et al., offers critical insight into the long-term consequences of SARS-CoV-2 infection in children and adolescents (1). By recruiting over 1,100 participants across 12 Italian centers and following them for 12 months, authors provide one of the most comprehensive pediatric long COVID datasets available to date. Using a standardized follow-up at 1–3, 3–6, and 6–12 months post-infection, and a child-adapted definition of long COVID aligned with World Health Organization (WHO) criteria, the study confirms that approximately 16% of infected children report persistent symptoms lasting over two months. These symptoms include respiratory complaints, fatigue, cognitive difficulties, sleep disturbances, and gastrointestinal issues. Importantly, the study reveals that while most children recover within a year, a significant subset continues to experience symptoms impacting their quality of life (1).

This well-designed multicenter study, with standardized outcomes and a control group, reveals that adolescents and females are especially vulnerable to neuropsychological sequelae. Despite lacking objective tests, it significantly advances understanding of pediatric long COVID (1).

However, despite the growing body of literature, a standardized follow-up protocol for children with suspected long COVID is still lacking. In this context, we would like to contribute to the discussion by sharing findings from two recent investigations conducted by our multidisciplinary team of pediatricians and child neuropsychiatrists at the Pediatric Clinic of the University of Campania “Luigi Vanvitelli” in Naples, Italy.

**Figure 1:**
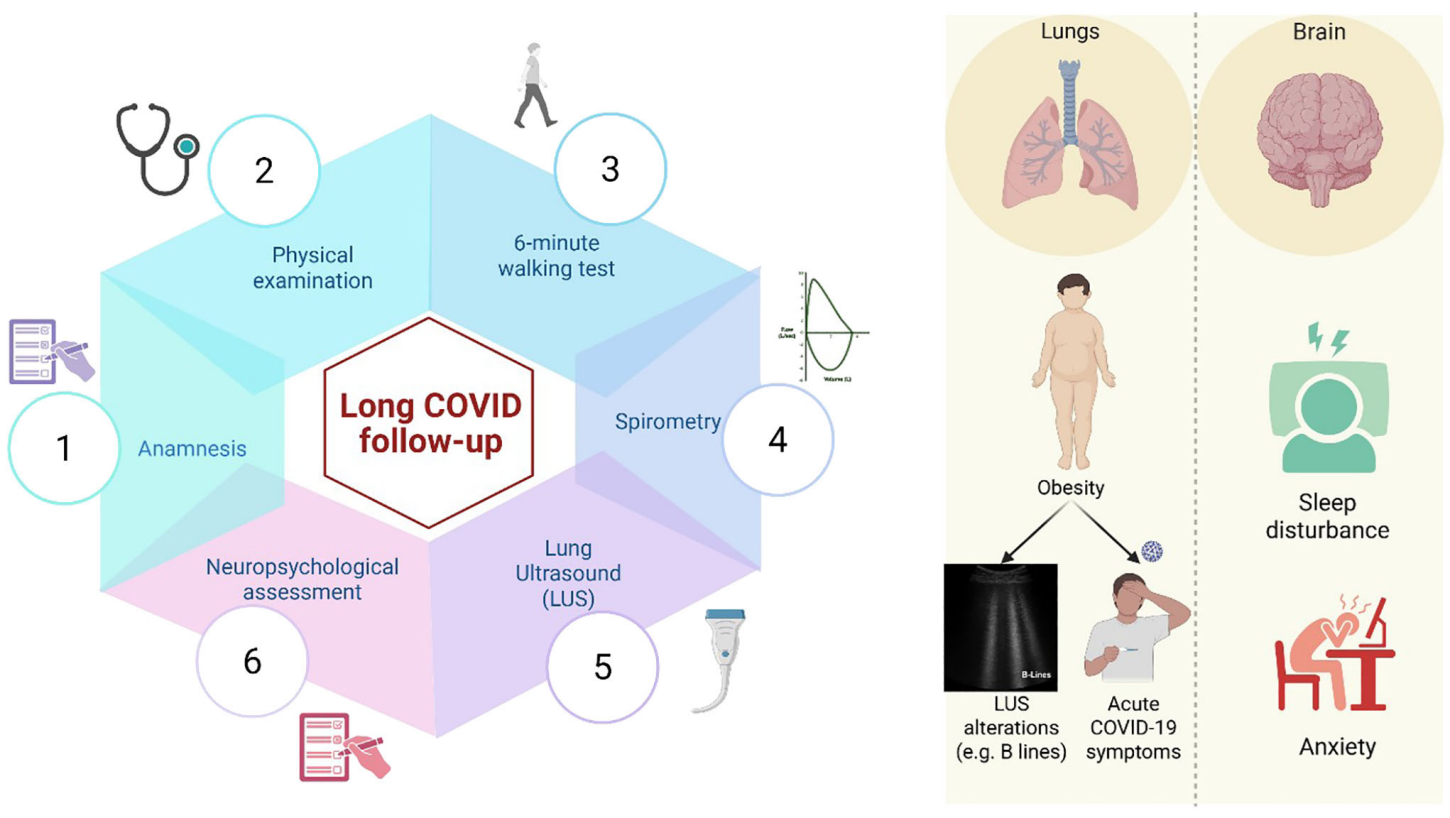
The figure illustrates the long COVID follow-up conducted at our Pediatric Clinic at the University of Campania “Luigi Vanvitelli” in Naples, Italy. We collected detailed medical histories, including SARS-CoV-2-related manifestations, conducted clinical examinations, a 6-minute walking test, spirometry, lung ultrasound (LUS), and a neuropsychological evaluation. Our findings confirmed that the most common long COVID symptoms in the pediatric population involve neuropsychological aspects and pulmonary manifestations. In particular, obesity was significantly associated with both the severity of the acute phase of the disease and the presence of LUS abnormalities. We also observed an increase in behavioral and sleep disturbances, especially among adolescents, with a broader impact on their physical and emotional well-being. These findings highlight the need for long-term, in-depth follow-up over the coming years. Figure created in https://BioRender.com.

We implemented a structured follow-up protocol for all children presenting with persistent symptoms following confirmed SARS-CoV-2 infection. This included a clinical examination, spirometry, a six-minute walking test, and lung ultrasound (LUS), a non-invasive and radiation-free technique, to identify subclinical pulmonary abnormalities and assess their relationship with clinical and anthropometric parameters. We also evaluated psychological symptoms using two validated questionnaires: the Child Behavior Checklist (CBCL) and the Sleep Disturbance Scale for Children (SDSC) **Figure 1**.

In our first study, we evaluated respiratory outcomes in a cohort of 104 children and adolescents with persistent symptoms beyond four weeks after SARS-CoV-2 infection. Using LUS, we identified abnormal findings such as pleural line irregularities and coalescent B-lines in 27% of patients. These findings were not associated with a history of asthma or allergic disease/sensitization, but showed a significant correlation with overweight status (BMI >85th percentile). This suggests that obesity may not only increase the risk of acute COVID-19 complications but also predispose to subtle, long-lasting pulmonary alterations in the post-acute phase (2).

Our second study focused on the neuropsychiatric impact of long COVID, analyzing behavioral and sleep disturbances through the CBCL and SDSC in a matched case-control cohort of 107 children and adolescents. We found a significant increase in internalizing symptoms, particularly anxiety, depression, and somatic complaints in post-COVID cases compared to controls. These behavioral changes were paralleled by a higher prevalence of sleep disturbances, notably sleep-disordered breathing and alterations during the sleep-wake transition phase. A strong correlation emerged between behavioral and sleep domain scores, underscoring the intertwined nature of emotional well-being and sleep quality (3).

These findings suggest that long COVID extends beyond physical manifestations, significantly affecting children’s emotional well-being, sleep patterns, and social functioning. These alterations are likely supported by neuroinflammatory mechanisms, sustained immune activation, or the cumulative impact of psychosocial stressors experienced during and after infection. Disrupted peer relationships, decreased school attendance, and a persistent sense of health-related uncertainty can amplify emotional distress and contribute to the emergence of behavioral issues, particularly in vulnerable populations such as adolescents. These holistic consequences emphasize the urgent need to reframe pediatric care, shifting from a purely somatic approach toward comprehensive, multidisciplinary follow-up strategies. Such protocols should be designed to monitor and address both the immediate and long-term neurodevelopmental, emotional, and social sequelae associated with pediatric long COVID (4, 5).

We thank the authors for their valuable contribution and for encouraging continued dialogue on pediatric COVID-19. We hope our clinical experience will inform future research and improve management strategies for long COVID. In particular, we believe that our structured follow-up protocol and the demonstrated utility of LUS may support efforts to standardize long-term care in affected children.
